# A Freezing-Thawing Damage Characterization Method for Highway Subgrade in Seasonally Frozen Regions Based on Thermal-Hydraulic-Mechanical Coupling Model

**DOI:** 10.3390/s21186251

**Published:** 2021-09-17

**Authors:** Qingsong Deng, Xiao Liu, Chao Zeng, Xianzhi He, Fengguang Chen, Siyu Zhang

**Affiliations:** 1 Three Gorges Research Center for Geo-Hazards of Ministry of Education, China University of Geosciences (Wuhan), Wuhan 430074, China; Dengqingsong@cug.edu.cn (Q.D.); zhangsiyujia@163.com (S.Z.); 2 China Communications Construction Company Second Highway Consultants Co., Ltd., Wuhan 430056, China; caozhen_shc@163.com (C.Z.); hexzh@126.com (X.H.); chenfengguang31@163.com (F.C.)

**Keywords:** seasonally frozen soil, highway subgrade, thermal–hydraulic–mechanical coupling, frost heave, COMSOL multi-physics, freeze–thaw damage degree

## Abstract

Seasonally frozen soil where uneven freeze–thaw damage is a major cause of highway deterioration has attracted increased attention in China with the rapid development of infrastructure projects. Based on Darcy’s law of unsaturated soil seepage and heat conduction, the thermal–hydraulic–mechanical (THM) coupling model is established considering a variety of effects (i.e., ice–water phase transition, convective heat transfer, and ice blocking effect), and then the numerical solution of thermal–hydraulic fields of subgrade can be obtained. Then, a new concept, namely degree of freeze–thaw damage, is proposed by using the standard deviation of the ice content of subgrade during the annual freeze–thaw cycle. To analyze the freeze–thaw characteristics of highway subgrade, the model is applied in the monitored section of the Golmud to Nagqu portion of China National Highway G109. The results show that: (1) The hydrothermal field of subgrade has an obvious sunny–shady slopes effect, and its transverse distribution is not symmetrical; (2) the freeze–thaw damage area of subgrade obviously decreased under the insulation board measure; (3) under the combined anti-frost measures, the maximum frost heave amount of subgrade is significantly reduced. This study will provide references for the design of highway subgrades in seasonally frozen soil areas.

## 1. Introduction

Frozen soil is a kind of soil containing ice at temperatures below 0 °C. It can be di-vided into permafrost and seasonally frozen soil if a seasonal freezing period exists. Seasonally frozen areas are widely distributed, accounting for 53% of the world’s land area, while in China, they account for 53.5% of the total land area [1,2,3]. China’s Belt and Road Initiative increased infrastructure construction in the Qinghai-Tibet seasonally frozen regions where multiple highway projects are planned [4,5]. In the process of cyclical freeze–thaw of subgrade due to periodic temperature changes, the road pavement located in seasonally frozen areas is susceptible to damage, which poses a great threat to the safety of highway transportation [6,7].

The mechanism of frost heave and thaw settlement of subgrade in seasonally frozen ground is complex. The heat transfer, moisture migration, and phase change all interact during the freeze–thaw process. This coupling effect is an important cause of subgrade freeze–thaw damage [8,9,10,11,12,13]. In order to effectively control subgrade damage and maintain the long-term safety of the highway in seasonally frozen regions, it is necessary to study the coupling mechanism of physical fields inside frozen soil, and to reveal the freeze–thaw law of subgrade in seasonally frozen soil areas.

In the research of railway subgrade in seasonally frozen soil regions, based on the field monitoring data of high-speed railway subgrade in seasonally frozen areas, Liu et al. [14,15] established the temperature field control equation considering the influence of phase change and moisture content, simulated the temperature field of subgrade under various subgrade fillings and different construction time, analyzed the change of subgrade freezing depth, studied the thermal state of culvert transition section of high-speed railway subgrade, and compared the influence of culvert geometry and antifreeze measures on temperature field. Yuan et al. [16] used ANSYS finite element software to simulate the temperature field of subgrade in the next 30 years according to the temperature boundary conditions of cement pavement, black pavement and thermal insulation black pavement of high-speed railway subgrade in seasonally frozen soil areas. The frost depth, pavement freezing index, subgrade thawing area and future trend were compared and analyzed. Tai et al. [17] used the finite element software COMSOL combined with the frost heave theory to develop the hydrothermal and deformation coupling analysis of high-speed railway foundation, and carried out a simulation and comparative analysis of a variety of anti-frost measures. Based on the classical hydrodynamic model and thermoelasticity theory, Zhang et al. [18] proposed the relational equation according to the soil–water characteristic curve and solid–liquid ratio, established a hydrothermal coupling model considering the effects of ice–water phase transition and temperature change on moisture migration, and then simulated and analyzed the frost heave deformation characteristics of high-speed railway subgrade. Zhang et al. [19] established a three-dimensional model of frozen soil subgrade based on finite element method, and analyzed the influence of heating hole spacing and depth on the temperature field of seasonally frozen soil railway subgrade.

In terms of research on highway subgrade in seasonally frozen soil regions, Li et al. [20] established the thermal–hydraulic-mechanical coupling model to simulate and analyze the highway subgrade in seasonal frozen soil area, and compared the deformation and ground temperature changes of anti-freezing cushion subgrade and original subgrade. Wang et al. [21] simulated the change of subgrade temperature field and the deformation characteristics of highway subgrade caused by temperature difference based on the actual climate in seasonally frozen soil areas, and analyzed the difference between sunny and shady slopes of subgrade. Zhang et al. [22] put forward XPS insulation board and rubber particles and fly ash modified soil to form a cold resistance layer, and then using finite element method to analysis the anti-freeze effect of different measures. Zhang et al. [23] analyzed the frost heave displacement of highway in seasonally frozen soil areas under the action of the sunny–shady slopes effect based on heat transfer theory and elastoplastic deformation theory. Based on the hydrothermal coupling model, Liu et al. [24] analyzed the influence of geosynthetic clay liner on the temperature field and water field of highway subgrade in seasonal frozen soil regions.

However, as Table 1 shows, this existing referenced literature has been rarely reported a simple and effective method to quantitatively evaluate the degree of damage of highway subgrade caused by the freeze–thaw cycle effect. In addition, there are few studies on anti-frost heave simulation of highway subgrade in seasonally frozen regions and the influence of extreme weather. It is noted that most studies on multi-field coupling simulation of highway subgrade in seasonally frozen soil areas only consider the comparison between numerical model and monitoring data. In fact, if the numerical simulation method is combined with other methods (e.g., physical models [25], data-driven [26], machine learning [27]), the accuracy of the simulation can be steadily improved. Therefore, further research on highway subgrade in seasonally frozen regions needs to be strengthened.

In this study, a new concept, namely freeze–thaw damage degree of subgrade, was defined by standard deviation of ice content in annual freeze–thaw cycle, so as to quantitatively evaluate and predict the vulnerable area in subgrade. From the perspective of this new concept, many phenomena and characteristics obtain new explanations and characterization methods (e.g., how does the sunny–shady slope effect quantitatively affect the damage degree of subgrades, and how to evaluate performance of various anti-frost measures from the new index of damage degree?). The purpose of this study is to put forward a methodology based on actual cases, and to provide references for the design of highway subgrades in a seasonally frozen regions.

## 2. Methodology

### 2.1. Technical Roadmap

**Figure 1:**
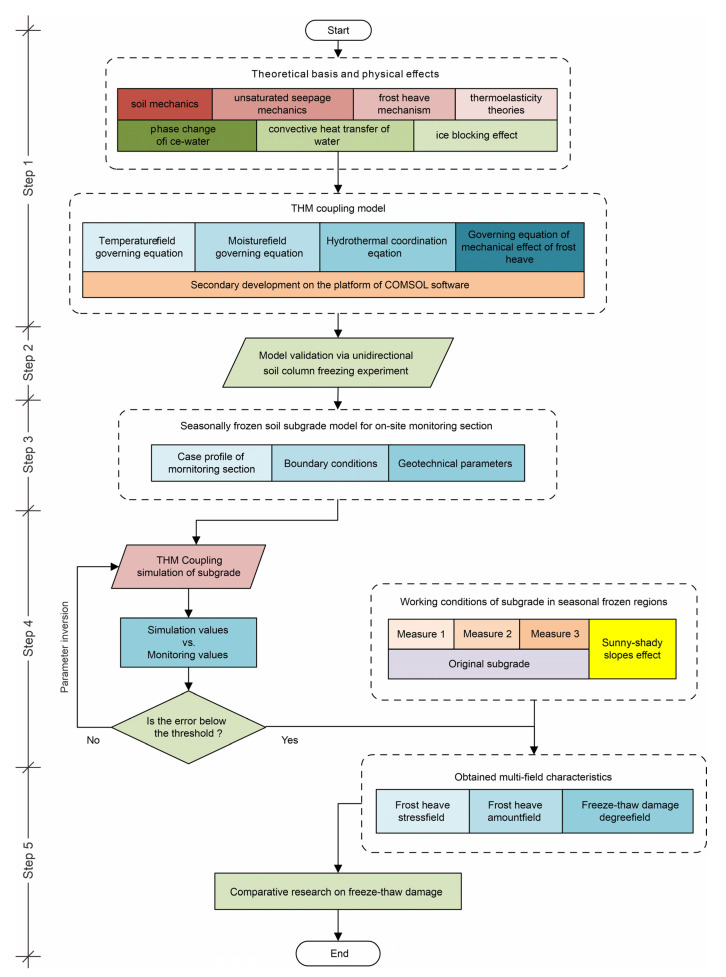
Technology roadmap.

The overall technical roadmap of this study is shown in Figure 1. First, the THM coupling equations considering phase change of ice–water, convective heat transfer of water, and ice blocking effect were established by secondary development on mathematical modules of COMSOL finite element software. Essentially, the THM coupling model established is a calculation engine used to generate multi-fields data for subsequent analysis. Therefore, the correctness of the model is of importance. In order to verify the effectiveness of the THM coupling model, a validation test is carried out by simulating the standard physical experiment. Then, the proven THM coupling model is run as the data-driven engine. A new concept, namely freeze–thaw damage degree of subgrade, was defined by standard deviation of ice content in the annual freeze–thaw cycle, so as to quantitatively evaluate and predict the vulnerable area in subgrade. In this way, various time-varying coupling fields (i.e., temperature, moisture, stress, frost heave amount, and freeze–thaw damage degree) were obtained and used to reveal the characters of highway subgrade in seasonally frozen regions. In addition, by applying different working conditions (i.e., anti-frost measures, extreme cold weather, and sunny–shady slopes effect), the differences in characteristics of subgrade freeze–thaw damage are analyzed.

As shown in Figure 1, there are several key steps.

- Step 1: Combining the soil mechanics, unsaturated seepage mechanics, frost heave mechanism, and thermoelasticity theories, and considering the ice–water phase transition, convective heat transfer and ice blocking effect, the THM coupling model was established and realized by the secondary development on the platform of COMSOL software.
- Step 2: Apply the THM coupling model to the unidirectional soil column freezing experiment, and then verify the effectiveness of the THM coupling model by comparing the simulated value with the experimental value.
- Step 3: Taking a monitoring section of subgrade in the Golmud to Nagqu portion of China National Highway G109) as a case study, select appropriate boundary conditions and geotechnical parameters to establish a seasonally frozen soil subgrade model.
- Step 4: Carry out THM coupling simulation of the subgrade model, and compare the simulation values and monitoring values on the spatial-temporal distribution. If the error exceeds the threshold, adjust the model until the response of THM model matches well with the on-site monitoring data through remote transmission.
- Step 5: Obtain multi-field characteristics (i.e., frost heave stress field, frost heave amount field, and freeze–thaw damage degree field) of the subgrade under different working conditions (i.e., three types of anti-frost measures, and the sunny-shady slopes effect) based on THM coupling model, and then conduct comparative research on freeze–thaw damage of the subgrade.

### 2.2. Governing Equations of THM Coupling Model

According to the seasonal frost characteristics, the following four statements are assumed: (1) the subgrade soil is homogeneous, isotropic, and incompressible; (2) only the flow of un-frozen water in the subgrade is considered, and the position of the ice is assumed to not change; (3) the influence of water vapor on the migration of unfrozen water, heat transfer, and the temperature change caused by liquid–gas phase transition are all neglected; (4) and only the latent heat of ice–water phase transition is considered as an internal heat source. Based on these assumptions, the THM coupling model of subgrade in seasonally frozen ground is established.

#### 2.2.1. Governing Equation of the Temperature Field

According to the conservation of energy and Fourier’s law of thermal conduction, taking the Harlan model [28] developed on fluid dynamics as the theoretical basis, regarding the latent heat of ice–water phase transition as the internal heat source, and considering the influence of unfrozen water flow on heat transfer, the two-dimensional partial differential equation of heat conduction is established:

(1) $$C\left(\theta \right)\frac{\partial T}{\partial t}=\frac{\partial }{\partial x}\left(\lambda \left(\theta \right)\frac{\partial T}{\partial x}\right)+\frac{\partial }{\partial y}\left(\lambda \left(\theta \right)\frac{\partial T}{\partial y}\right)+L\rho_{i}\frac{\partial \theta_{i}}{\partial t}-\rho_{w}C_{w}\left(v_{x}\frac{\partial T}{\partial x}+v_{y}\frac{\partial T}{\partial y}\right)$$

where T denotes temperature (°C), t is time (s), C(θ) represents volume heat capacity of soil ($J/m^{3}$) and $\lambda \left(\theta \right)$ is thermal conductivity ($W\cdot m^{-1}\cdot K^{-1}$), both are functions of water content [29], L is latent heat of ice–water phase transition, which is 334,560 J/kg in general engineering thermal calculation, $\rho_{i}$ and $\rho_{w}$ represent density of ice and water, respectively (kg·m^3^), $C_{w}$ is specific heat of water, which is 4180 J·kg^−1^·K^−1^, $\theta_{i}$ is volumetric ice content, and v is the liquid water flux (m/s).

#### 2.2.2. Governing Equation of the Moisture Field

According to the Gibbs–Thomson effect [30,31,32,33], there is still unfrozen water inside the subgrade even in a frozen zone at a temperature that is below the bulk freezing point of water (0 °C), and the unfrozen water in either the frozen zone or the thawing zone meets Darcy’s law. Based on the Richards equation [34], considering the blocking effect of pore ice in frozen soil, the governing equation of water transport in unsaturated soil with unfrozen water content as an independent variable can be expressed as [35]:

(2) $$\frac{\partial \theta_{u}}{\partial t}=\frac{\partial }{\partial }\left(D\left(\theta_{u}\right)\frac{\partial \theta_{u}}{\partial x}\right)+\frac{\partial }{\partial y}\left(D\left(\theta_{u}\right)\frac{\partial \theta_{u}}{\partial y}+k{\left(\theta_{u}\right)}_{y}\right)-\frac{\rho_{i}}{\rho_{w}}\cdot \frac{\partial \theta_{i}}{\partial t}$$

where $\theta_{u}$ is volumetric content of unfrozen water, and the total volumetric water content is expressed as $\theta =\theta_{u}+\rho_{i}/\rho_{w}\cdot \theta_{i}$. $k{\left(\theta_{u}\right)}_{y}$ represents permeability coefficient of unsaturated soil in gravity direction (m/s), $D\left(\theta_{u}\right)$ denotes water diffusion coefficient ($m^{2}/s$).

Based on the Buckingham–Darcy law and the fluid continuity equation, the liquid water flux can be expressed as [36,37]:

(3) $$v=-\frac{K_{r}K}{\eta_{w}}\left(\nabla P_{w}+\rho_{w}g\nabla y\right)$$

where $K_{r}$ is the relative permeability of the liquid, K represents the inherent permeability of unsaturated soil ($m^{2}$), $P_{w}$ represents the pore water pressure (Pa), g is the acceleration of gravity ($m/s^{2}$), y is the gravity potential (m), $\eta_{w}$ denotes the dynamic viscosity of liquid water ($N\cdot s/m^{2}$) and the dynamic viscosity coefficient is usually a function of absolute temperature [38].

This paper only considers the seepage of liquid water without considering the influence of other liquid solutions. Therefore, the relative permeability $K_{r}$ of liquid is ignored. According to unsaturated soil mechanics [39], the inherent permeability and pressure head can be expressed as:

(4) $$K=\frac{k\left(\theta_{u}\right)\eta_{w}}{\rho_{w}g}$$

(5) $$h=\frac{P_{w}}{\rho_{w}g}$$

where $k\left(\theta_{u}\right)$ is the permeability coefficient of unsaturated soil (m/s), h denotes the pressure head (m).

According to Equations (3)–(5), we can obtain the expression:

(6) $$v=-k\left(\theta_{u}\right)\nabla (h+y)$$

Since $D\left(\theta_{u}\right)=k\left(\theta_{u}\right)/c\left(\theta_{u}\right)$ and $c\left(\theta_{u}\right)=\partial \theta_{u}/\partial h$, Equation (6) can be expressed as:

(7) $$v=-D\left(\theta_{u}\right)\nabla \theta_{u}-k{\left(\theta_{u}\right)}_{y}$$

where $c\left(\theta_{u}\right)$ is specific water capacity ($m^{-1}$). It should be noted that some parameter values (viscosity parameters $\eta_{w}$, etc.) in Equation (3) will not be involved in this paper. These values are contained in $k\left(\theta_{u}\right)$, $c\left(\theta_{u}\right)$ and $D\left(\theta_{u}\right)$ by transforming form, and their specific form will be given in Equations (23)–(25) below.

#### 2.2.3. Hydrothermal Coordination Equation

Regarding the simultaneous equations of the governing equations of temperature field and moisture field, there exist three variables (i.e., temperature, volumetric content of unfrozen water, and volumetric ice content). Therefore, a relationship between the three variables needs to be introduced to solve the hydrothermal coupling equations simultaneously. Here, a concept of solid–liquid ratio in introduced by Bai et al. [40] to assume the volumetric ice content and volumetric content of unfrozen water meet a certain ratio in the frozen zone:

(8) $$B_{i}\left(T\right)=\frac{\theta_{i}}{\theta_{u}}=\left\{\begin{array}{cc} \frac{\rho_{w}}{\rho_{i}}\left({\left|\frac{T}{T_{f}}\right|}^{b}-1\right) & \left(T<T_{f}\right)\\ 0 & \left(T⩾T_{f}\right) \end{array}\right.$$

where $T_{f}$ is the subgrade freezing temperature (°C), which is related to soil type and salt content. b can be determined from experience with the value of 0.56 for clay, 0.47 for silt, and 0.61 for sand and gravel. Then, volumetric ice content of ice is expressed as:

(9) $$\theta_{i}=B_{i}\left(T\right)\cdot \theta_{u}$$

#### 2.2.4. Governing Equations of Mechanical Effect of Frost Heave

In this paper, the mechanics governing equations in this article are developed on the basis of the classic linear elastic theory [41]. The frost heave stress is defined as the expansion stress on the surrounding soil when phase changes from water to ice at its bulk freezing point, that is the product of thermal expansion strain and volume modulus:

(10) $$P_{frozen}=K_{T}\varepsilon_{vol}{}^{T}=K_{T}\left(\varepsilon_{x}{}^{T}+\varepsilon_{y}{}^{T}+\varepsilon_{z}{}^{T}\right)=\frac{E_{T}\left(\varepsilon_{x}{}^{T}+\varepsilon_{y}{}^{T}+\varepsilon_{z}{}^{T}\right)}{3\left(1-2\mu_{T}\right)}$$

where $P_{frozen}$ is frost heave stress (MPa), $K_{T}$ is bulk modulus (MPa), $E_{T}$ is Young’s modulus of road foundation filling (MPa), $\mu_{T}$ is Poisson’s ratio, $\varepsilon_{vol}{}^{T}$ is thermal expansion volumetric strain, $\varepsilon_{x}{}^{T}$, $\varepsilon_{y}{}^{T}$ and $\varepsilon_{z}{}^{T}$ represent thermal expansion linear strain in three axes directions, respectively. For two-dimensional homogeneous isotropic material, there exist $\varepsilon_{z}{}^{T}=0$ and $\varepsilon_{x}{}^{T}=\varepsilon_{y}{}^{T}$. Therefore, a uniform value $\varepsilon_{th}$, namely thermal expansion linear strain, could be introduced, and then Equation (10) is further expressed as below.

(11) $$P_{frozen}=\frac{2E_{T}\varepsilon_{th}}{3\left(1-2\mu_{T}\right)}$$

The Young’s modulus and Poisson’s ratio of subgrade soil both vary with temperature [42], given as follows:

(12) $$E_{T}=\left\{\begin{array}{cc} A_{1}{+B}_{1}{\left|T\right|}^{0.6} & \left(T<T_{f}\right)\\ A_{1} & \left(T⩾T_{f}\right) \end{array}\right.$$

(13) $$\mu_{T}=\left\{\begin{array}{cc} A_{2}+B_{2}\left|T\right| & \left(T<T_{f}\right)\\ A_{2} & \left(T⩾T_{f}\right) \end{array}\right.$$

where $A_{1}$, $A_{2}$, $B_{1}$ and $B_{2}$ are the parameters related to soil. For the implementation process by COMSOL, T is derived from the calculated results of hydrothermal field and then imported into COMSOL to calculate $E_{T}$ and $\mu_{T}$ based on Equations (12) and (13).

The uniform linear strain induced by ice–water phase transition (i.e., $\varepsilon_{th}$) [43] is calculated as follows:

(14) $$\varepsilon_{th}=\alpha \left(T-T_{ref}\right)$$

where α is thermal expansion coefficient, $T_{ref}$ is the reference temperature of thermal expansion (°C), and here is the freezing temperature. The temperature difference $\left(T-T_{ref}\right)$ is fixed at 1, which aims to fix the relationship between temperature and strain.

Further, the thermal expansion coefficient is calculated as below:

(15) $$\alpha =\sqrt[{3}]{\eta \left(\theta_{i}\right)+1}-1$$

where $\eta \left(\theta_{i}\right)$ is frost heave coefficient, which is changed by volumetric ice content in the soil and also affected by the difference of soil types. Reference [17] proposed a semi-empirical formula to determine $\eta \left(\theta_{i}\right)$ as below:

(16) $$\eta {\left(\theta_{i}\right)}_{1}=\left\{\begin{array}{cc} 0.076\frac{\rho_{i}}{\rho_{d}}\theta_{i}-0.0028 & \left(\theta_{i}>0.037\frac{\rho_{d}}{\rho_{i}}\right)\\ 0 & \left(\theta_{i}⩽0.037\frac{\rho_{d}}{\rho_{i}}\right) \end{array}\right.$$

(17) $$\eta {\left(\theta_{i}\right)}_{2}=\left\{\begin{array}{cc} 0.156\frac{\rho_{i}}{\rho_{d}}\theta_{i}-0.0042 & \left(\theta_{i}>0.027\frac{\rho_{d}}{\rho_{i}}\right)\\ 0 & \left(\theta_{i}⩽0.027\frac{\rho_{d}}{\rho_{i}}\right) \end{array}\right.$$

(18) $$\eta {\left(\theta_{i}\right)}_{3}=\left\{\begin{array}{cc} 0.089\frac{\rho_{i}}{\rho_{d}}\theta_{i}-0.0003 & \left(\theta_{i}>0.003\frac{\rho_{d}}{\rho_{i}}\right)\\ 0 & \left(\theta_{i}⩽0.003\frac{\rho_{d}}{\rho_{i}}\right) \end{array}\right.$$

where $\eta {\left(\theta_{i}\right)}_{1}$, $\eta {\left(\theta_{i}\right)}_{2}$, $\eta {\left(\theta_{i}\right)}_{3}$ are frost heave coefficients of coarse sand, crushed stone and silty sand, respectively. For the implementation process by COMSOL, the interpolation of volumetric ice content calculated by hydrothermal coupling is imported into the thermal expansion coefficient interface. In this way, a standard thermal expansion model in COMSOL is revised to match the properties of frozen soil, so that the frost heave stress is applied as a stress field on the subgrade, which results in frost heave deformation.

It must be pointed out that from the solution of the above THM model, the influence of temperature field and moisture field on the stress field is unidirectional. That is, in this paper: (1) the change of soil porosity caused by the changed stress field is not considered, so the effect of stress on seepage is ignored; (2) the temperature fluctuation caused by the changed stress field is also not considered. Although it is an incompletely coupled algorithm between mechanical field and hydrothermal field, it can still be classified as a generalized THM coupled algorithm [44,45].

### 2.3. New Concepts to Quantify Freeze–Thaw Damage of Subgrade in Seasonally Frozen Regions

#### 2.3.1. Definition of the Amount of Frost Heave of Subgrade

At present, there is no strict and unified definition for quantitative description of frost heave. This article enumerates two kinds of representative points of view.

The first definition, as shown in reference [46], is that the frost heave deformation is mainly caused by the volume expansion during the phase transition from water to ice. When the volumetric ice content exceeds the critical value, the soil will experience frost heave deformation, and the frost heave amount is the excess ice volume (dimension [L^3^]). Under this conceptual model, frost heave amount is the integral of the volume strain generated by the liquid–solid phase transition in the spatial region:

(19) $$V_{frozen}=\int_{\Omega }\left(\varepsilon_{x}{}^{T}+\varepsilon_{y}{}^{T}+\varepsilon_{z}{}^{T}\right)dV$$

where $V_{frozen}$ is frost heave amount, $\varepsilon_{x}{}^{T}$, $\varepsilon_{y}{}^{T}$ and $\varepsilon_{z}{}^{T}$ represent thermal expansion linear strain in three axes directions, respectively, V denotes volume, and Ω is space region integral calculation. For two-dimensional homogeneous isotropic material, the Equation (19) is further expressed as below:

(20) $$V_{frozen}=2\int_{\Omega }\varepsilon_{th}dV$$

where $\varepsilon_{th}$ represents thermal expansion linear strain.

The second definition is derived from engineering practice, and the amount of frost heave is defined as the height difference of the subgrade surface before and after freezing (dimension [L]).

Although both definitions can describe the overall deformation after the occurrence of frost heave, they are different in dimension. The former is measured by volume, while the latter is measured by length. The more important defect is that both definitions do not support the concept of “field”, which is not conducive to describing the frost heave deformation from the perspective of spatial variation. Therefore, this paper defines the point’s frost heave as the vertical displacement of the point before and after freezing (dimension [L]). Thus, the frost heave has the concept of “field”, and its value on the subgrade surface (i.e., the vertical displacement of the subgrade surface) is consistent with the aforementioned second definition.

In essence, the amount of frost heave is here defined as the deformation in the vertical direction. Note that the deformation of subgrade after construction comes from two effects. Firstly, the variation of the seepage field causes the change of stress, and this stress results in soil deformation. Secondly, the volume change during the phase transition of water in the soil affects the original stress field of the subgrade, which triggers a coordinated deformation of the subgrade to achieve a new balance. The first effect is secondary, and the frost heave deformation is primarily due to the latter one. In COMSOL, the first effect is calculated by considering the pore water pressure in the soil, and the second effect is calculated by the secondary development of the thermal expansion module of solid mechanics, which is built into COMSOL as shown in Section 2.2.4. From the perspective of COMSOL, under the above framework, the frost heave amount of any point (dimension [L]) is actually treated as solving the vertical displacement of the point via the thermal expansion interface in the COMSOL solid mechanics module.

#### 2.3.2. Definition of Freeze–Thaw Damage Degree of Subgrade

The subgrade damage is attributed to two aspects. The first one is the extrusion and cracking due to frost heave during the frost days, and the second one is the subsidence caused by the melting of ice in the subgrade during the frost-free days. It is very natural that both aspects can be characterized by the process of increasing and decreasing of ice content. During the period of annual freeze–thaw cycle, the more frequent and more drastic the change of the ice content in the subgrade is, the greater the inevitable damage will be. Frequent and drastic changes in the freeze–thaw cycle cause the ice content, as a statistic variable, to increase its dispersion.

Considering that standard deviation is of importance to characterize the degree of statistical dispersion, this paper extracts the mass ice content of subgrade every 24 h during one year’s complete freeze–thaw cycle season and calculates its standard deviation to quantify the fluctuation of ice content, so as to quantify the damage degree suffered by subgrade. The area with the higher standard deviation of volumetric ice content indicates the higher damage in the corresponding location within the statistical period, i.e., one year. Moreover, due to the expected repeatability of annual seasonal cycles, the damage will be accumulated along with the repeated cycles over the years to come. Thus, although the relative magnitude of damage between different areas remains the same, the damage degree in each area tends to keep growing during the full life of the project operation.

By calculating the degree of freeze–thaw damage in each area of subgrade, a field of damage degree can be obtained to quantify the freeze–thaw cycle effect on the subgrade. As a result, the high-risk area in subgrade can be predicted in advance, which is of great significance to engineering practice. This method is simpler and more intuitive than the general method (artificial monitoring, test analysis, etc.), which consumes less manpower and material resources and is easier to implement. It can provide a theoretical reference for the prevention of frost damage in seasonally frozen soil areas.

### 2.4. Model Validation

#### 2.4.1. Profile of Soil Column Freezing Experiment

The thermal cycle effect significantly affects the moisture distribution and mechanical behaviors of the soil [47,48,49,50]. In order to validate the effectiveness of the THM coupling model in Section 2.2, this paper simulated the existing indoor frozen soil column moisture migration test to carry out the inversion proof. Experimental data were obtained from the freezing test of the loam sampled from Zhangye, China, under closed system conducted by Hu [51,52].

**Figure 2:**
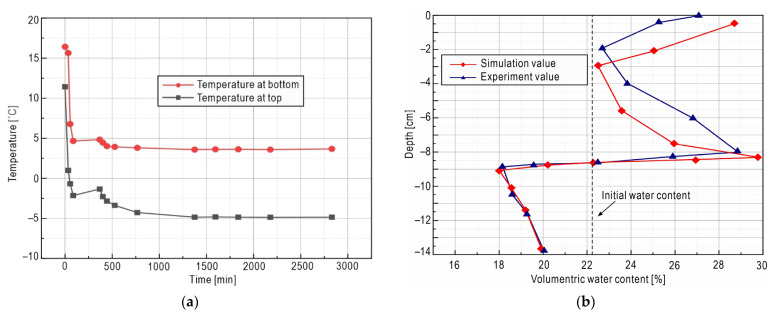
Result comparing. (**a**) Temperature boundary at the top and bottom of the sample; (**b**) total water content distribution at the end of freezing.

The cylindrical soil test sample is 13.68 cm high and 11.36 cm in diameter. The dry density is 1500 kg/m^3^, and the initial volumetric water content is 22%. The temperature at top and bottom of soil column as shown in Figure 2a, and the soil column side is wrapped with insulation material to make it adiabatic. The initial temperature of the sample is shown in Table 2. The soil column was frozen from top to bottom and the whole test was carried out under closed conditions, that is, there was no external water supplement or drain, and only water redistribution within the test sample occurred.

#### 2.4.2. Parameter Setting

It is noted that some parameters in Equations (1) and (2) are not constants, but change with the temperature or water content of the soil. These parameters are mainly divided into thermal parameters and hydraulic characteristic parameters.

Thermal parameters include volumetric heat capacity and thermal conductivity, shown below [53]:

(21) $$C\left(\theta \right)=\rho \frac{C_{s}+\left(W-W_{u}\right)C_{i}+W_{u}C_{w}}{1+W}=\rho_{d}C_{s}+\rho_{w}\theta_{u}C_{w}+\rho_{i}\theta_{i}C_{i}$$

(22) $$\lambda \left(\theta \right)=\lambda_{s}{}^{\theta_{s}}\lambda_{w}{}^{\theta_{u}}\lambda_{i}{}^{\theta_{i}}$$

where ρ and $\rho_{d}$ are the natural density and dry density of soil, respectively, W and $W_{u}$ are the total moisture content and unfrozen moisture content of soil, respectively, $\theta_{s}$ is the volume content of soil, $\lambda_{w}$ represents the thermal conductivity of water, $C_{i}$ and $\lambda_{i}$ are the specific heat and thermal conductivity of ice, respectively, $C_{s}$ denotes the soil specific heat ($J\cdot kg^{-1}\cdot K^{-1}$), and $\lambda_{s}$ is the soil thermal conductivity. Note that $C_{s}$ and $\lambda_{s}$ take different values in the range of frozen soil, thaw soil, and phase transition temperature. Therefore, both of them can be expressed by setting step function when calculating in COMSOL.

The seepage process in unsaturated frozen soil is complicated, and the hydraulic field involves many related parameters. This paper mainly analyzes the hydraulic field based on van Genuchten (VG) model [29,54,55,56,57]. The relevant parameters are as follows:

(23) $$D\left(\theta_{u}\right)=\frac{k\left(\theta_{u}\right)}{\left|c\left(\theta_{u}\right)\right|}$$

(24) $$k\left(\theta_{u}\right)=k_{s}S^{l}{\left(1-{\left(1-S^{1/m}\right)}^{m}\right)}^{2}$$

(25) $$c\left(\theta_{u}\right)=\frac{\partial \theta_{u}}{\partial h}=\frac{-am(\theta_{sa}-\theta_{re})S^{1/m}{\left(1-S^{1/m}\right)}^{m}}{\left(1-m\right)}$$

(26) $$S=\left(\theta_{u}-\theta_{re}\right)/\left(\theta_{sa}-\theta_{re}\right)$$

where $c\left(\theta_{u}\right)$ represents specific water capacity ($m^{-1}$) and $k\left(\theta_{u}\right)$ represents permeability coefficient of unsaturated soil (m/s), $k_{s}$ is saturated permeability coefficient of soil, a, m and l are empirical parameters of hydraulic characteristics, S is the relative saturation of soil, which is a linear function of volumetric unfrozen water content, $\theta_{re}$ and $\theta_{sa}$ are residual volumetric water content and saturated volumetric water content, respectively. Note that the permeability coefficient should be multiplied by I ($I=10^{-\theta_{i}}$) due to the blocking effect of pore ice in frozen area [58].

#### 2.4.3. Results Comparison of Numerical Simulation vs. Physical Experiment

The coefficient partial differential equations provided by COMSOL are modified to match the THM governing equations to simulate the test. Physical parameters used in the simulation were referred to the work of Li et al. [52], as shown in Table 3 and Table 4. The results comparison between numerical simulation and physical experiments are shown in Figure 2b.

Comparing volumetric water contents in different depths of the soil column, it can be seen from Figure 2b that the value in the unfrozen area (lower part) of the soil column is significantly reduced from the initial value before the test; however, the value in the frozen area (upper part) is substantially increased. This result is mainly due to the effect of temperature gradient. When the temperature gradient is relatively small, the freezing rate is relatively small, and the penetration speed of the freezing front is correspondingly slow, so that the water migration speed is greater than that of the freezing front invasion speed, and the moisture has enough time to migrate to the frozen area, resulting in the decrease of the water content in the unfrozen area and the increase of the water content in the frozen area. At a depth of about 8.7 cm in the soil column (i.e., freezing front), the volumetric water content presents a peak value due to the freezing of migrated water and the blocking effect of pore ice on part of the unfrozen water.

Comparing the results from physical experimental values and the numerical simulation results, the overall trend in Figure 2b is basically consistent, which indicates that the THM model is working with considerable precision to match the physical reality model. Once the THM model is verified, and the multi-field data obtained through it are reasonable, then the data-driven definitions in Section 2.3 and related further analysis are shown to be reliable.

## 3. Case Study

### 3.1. Profile of Subgrade Monitoring Section

**Figure 3:**
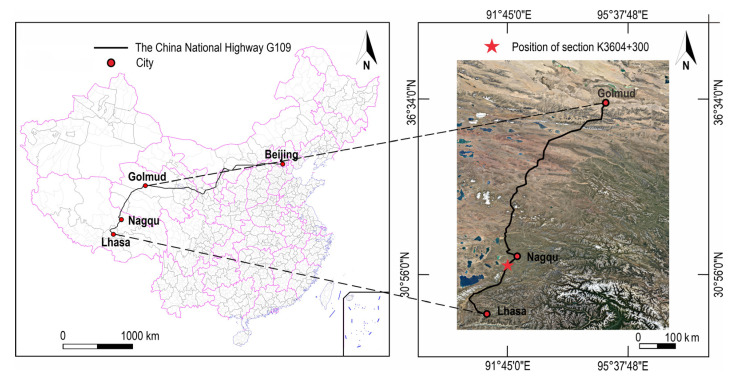
Location of the subgrade monitoring section K3604 + 300 of China National Highway G109 [59].

**Figure 4:**
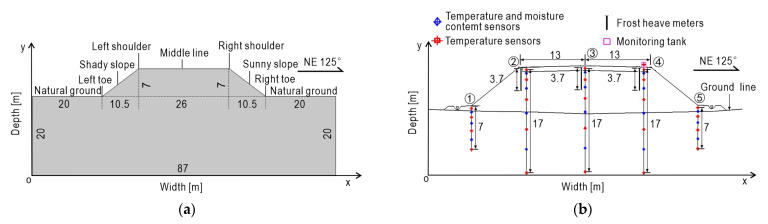
Schematic drawings of frozen soil subgrade. (**a**) Subgrade model size; (**b**) position of temperature, moisture and displacement sensors for frozen soil subgrade.

The Golmud-Nagqu highway is a portion of China National Highway G109 connecting Beijing and Lhasa. The subgrade section to be researched in this paper is located at mileage pile number K3604 + 300 of this highway in Xiangmao Township, Seni District, Nagqu City. The location of section K3604 + 300 is shown in Figure 3. The embankment is 26 m wide, 7 m high, and the slope ration is 1:1.5. The subgrade is mainly filled with sandy soil and then rolled. The left and right boundaries of the numerical model are 20 m range to the left and right toes of the subgrade, respectively, and the bottom boundary is 20 m downward from the natural ground line. The schematic diagram of the subgrade model is shown in Figure 4a.

### 3.2. Temperature Field Monitoring and Analysis

**Figure 5:**
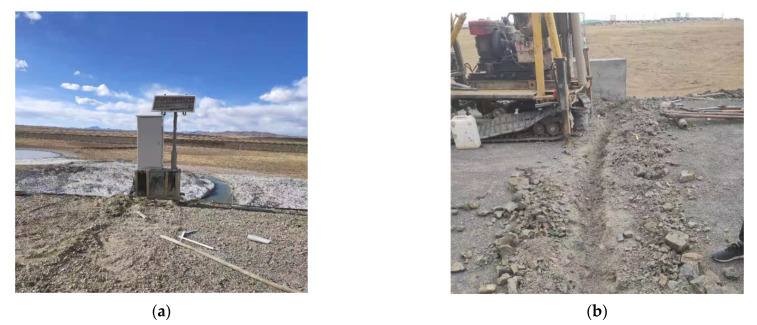
Field installation diagram of the instrument at section K3604 + 300. (**a**) Monitoring box and solar power supply module of section K3604 + 300; (**b**) wiring groove construction.

In order to obtain time-history of hydrothermal field and displacement field of the subgrade in seasonally frozen region, multiple sets of sensors including temperature sensors, moisture content sensors, and frost heave meters are installed at the monitoring subgrade section K3604 + 300. Sensors are set to be dense on the upper and sparse on the lower so as to keep balance performance between field coverage and data accuracy. The maximum buried depth is 17 m below the road surface. A total of 5 monitoring boreholes are set in the section, in which 32 temperature sensors, 18 water content sensors and 3 frost heave meters are installed at certain depths. Each borehole is set up with 6 to 9 temperature sensors of different depths and 3 to 4 water content sensors in different depths. Sensor layout is shown in Figure 4b, and its construction scene is shown in Figure 5.

**Figure 6:**
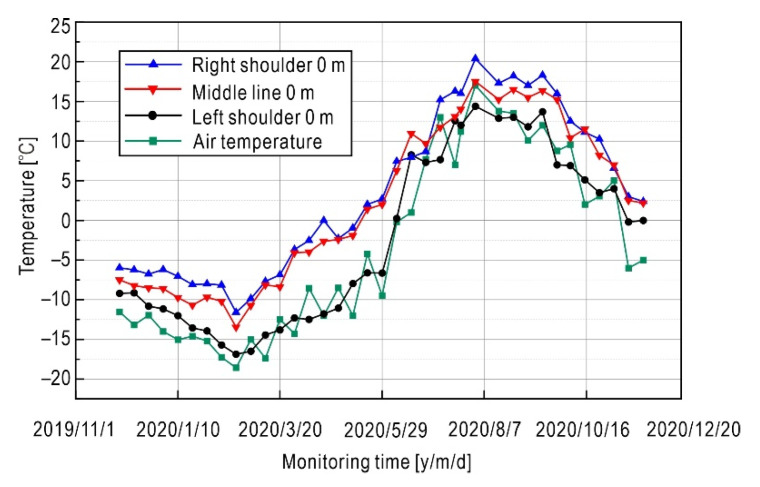
Ground temperature time-history of subgrade within a year. (The sensor at the depth of 0 m is not completely exposed to air, but buried in shallow soil close to the surface).

Real-time monitoring data were collected from various sensors by data acquisition modules and then transmitted to a remote server via the Internet for further analysis. In this way, the time-histories of THM fields are obtained. Figure 6 presents changes of ground temperature in different locations of the subgrade surface within a year. It is noted that the ground temperature of the left shoulder is lower than that of other positions of the subgrade, while the ground temperature of the middle line of the subgrade is close to that of the right shoulder. In February, the ground temperature of each location of the subgrade reaches the lowest value. The average ground temperature of the left shoulder in February is about −16°C, while the average temperature of the right shoulder is about −12 °C. The temperature difference between the left and right shoulder can reach about 4 °C. After March, it began to warm, and after May, it entered the warm season. In August, the temperatures of the subgrade reached their peaks up to about 20 °C, with a feature that the temperature of the right shoulder is higher than that of the middle line and the left shoulder. From March to May, the temperature difference is large, which is due to the possible interference by road construction during this period. In addition, the temperature difference between day and night in Nagqu is large and the temperature fluctuation is relatively large, so the temperature difference between ground and air does not always remain unchanged.

**Figure 7:**
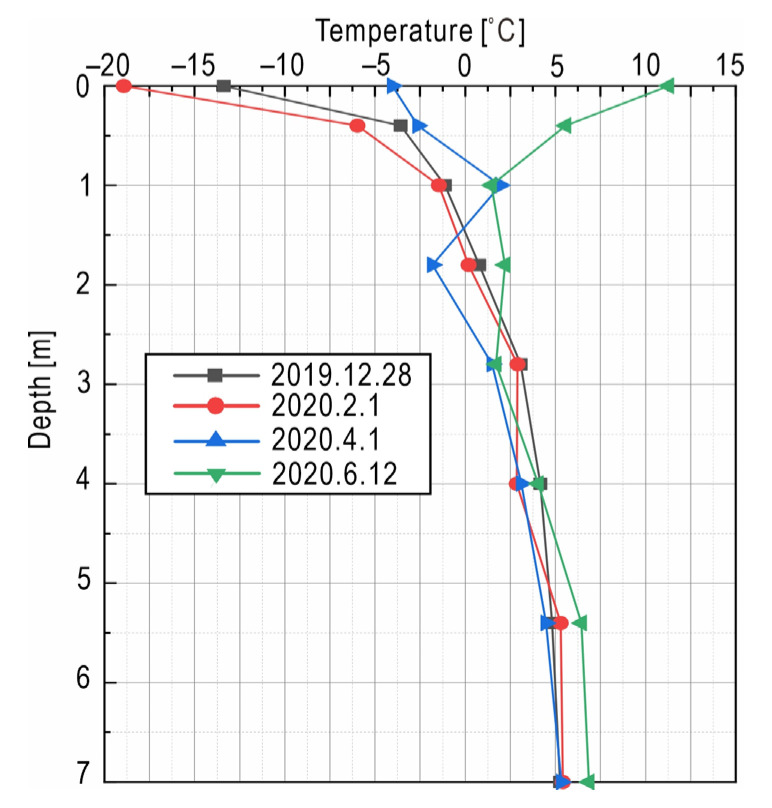
Ground temperature at the left toe of slope changing with depth on different days.

Field monitoring data can be used to find out the distribution of underground temperature with depth. Taking the left toe of the embankment as an example, Figure 7, illustrates the temperature–depth curve. Due to the influence of the external temperature of the air, the fluctuation of the temperature gradient of the shallow zone within a 4 m depth from the natural ground is relatively large, with the maximum fluctuation value of about 20 °C. However, at a depth exceeding 4 m, the influence of external air temperature is weak, and the temperature tends to be stable with limited gradient changes.

### 3.3. Boundary Conditions and Parameters of THM Coupling Model for Subgrade

By its very nature, the variation of multi-physical fields inside the subgrade is mainly driven by the change of external ambient temperature, so the setting of the temperature boundary is of particular importance. Based on the on-site monitoring data and the temperature setting strategy in reference [60,61,62,63], it is assumed that the subgrade temperature boundary approximately satisfies the sine function. In addition, the section of subgrade is mainly northwest to southeast direction (NE 125°), as shown in Figure 4, resulting in different solar radiation intensity on each side of the subgrade. Therefore, the so-called sunny–shady slopes effect [64,65] on the subgrade should be considered in the temperature boundary setting. Meanwhile, considering the impact of climate warming, the temperature of the Qinghai-Tibet Plateau is expected to rise by 2.6 °C in the following 50 years [66,67,68]. Therefore, the final temperature boundary is fitted by the following equation:

(27) $$T(t)=T_{0}+A_{0}\sin(\frac{2\pi t}{8640}+\varphi )+\frac{2.6t}{8640\times 50}$$

where $T_{0}$ is the average annual surface temperature (°C), $A_{0}$ denotes annual amplitude of ground temperature (°C), φ is initial phase angle, t represents time (h). According to the measured data from 2019 to 2020, the parameters of Equation (27) for temperature boundary formula at each surface position of the subgrade are shown in Table 5.

On the basis of laboratory tests and referring to relevant existing literature [18], the physical parameters of soil in each layer of the subgrade are shown in Table 6 and Table 7. Firstly, the subgrade above the natural ground was removed, the temperature of natural ground was taken as the boundary condition. Second, apply the THM coupling model to calculate the initial stable temperature field, and the calculation start time is August 1, 2019. According to the on-site monitoring data, the initial volumetric water content is set to 20%, and the temperature boundary on the left and right sides of the subgrade is zero flux boundary. The temperature gradient of the bottom boundary is 0.45 °C/m, which is equal to the heat flux boundary condition when multiplied by the corresponding heat conductivity coefficient. Since external water supply is not considered, the boundary around the field is taken as zero water flux. The boundary at the bottom of subgrade is set as constraint boundary, the boundary at both left and right sides of subgrade is set as roller support boundary, and the subgrade surface, slope and natural ground are set as free boundary. Therefore, when calculating the deformation, there is no displacement at the bottom of subgrade, and no lateral displacement occurs on the left and right sides of subgrade.

## 4. Result and Analysis

### 4.1. Hydraulic and Thermal Fields of Subgrade

#### 4.1.1. Spatial–Temporal Distribution of Temperature Field

The variation of subgrade temperature in the seasonally frozen region has obvious seasonal characteristics. In this paper, the calculation results on typical time points (i.e., 28 December 2019, 1 February 2020, 1 April 2020, and 12 June 2020) are selected for analysis.

**Figure 8:**
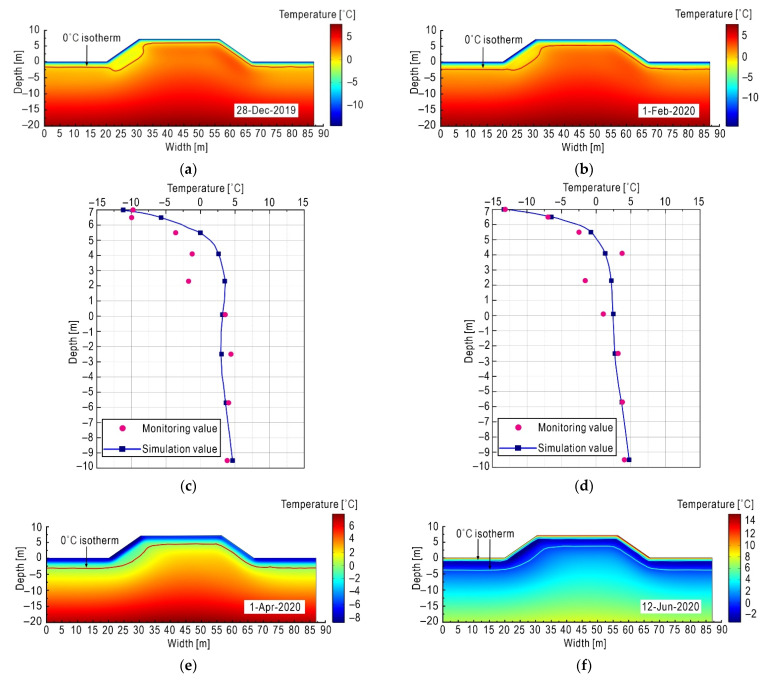

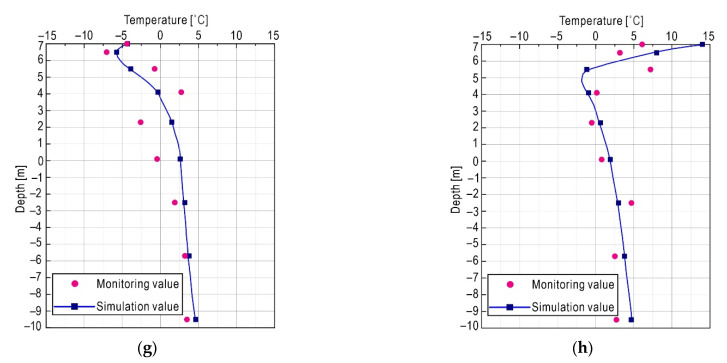
Subgrade temperature field at different time. (**a**) Simulated temperature contours on 28 December 2019; (**b**) simulated temperature contours on 1 February 2020; (**c**) temperature–depth curves of the middle line on 28 December 2019: simulated value versus on-site monitoring; (**d**) temperature–depth curves of the middle line on 1 February 2020: simulated value versus on-site monitoring; (**e**) simulated temperature contours on 1 April 2020; (**f**) simulated temperature contours on 12 June 2020; (**g**) temperature–depth curves of the middle line on 1 April 2020: simulated value versus on-site monitoring; (**h**) temperature–depth curves of the middle line on 12 June 2020: simulated value versus on-site monitoring.

As can be seen from Figure 8, the temperature changes dramatically in the shallow range below the pavement, and the range affected by external temperature changes is about 0~8 m below the surface, while the temperature is relatively stable in the area more than 8 m deep. When the external temperature is lower than the subgrade temperature inside, the heat is transferred from the subgrade inside to outside, causing the internal temperature of the subgrade to drop. As shown in Figure 8a, due to the short duration of freezing, the depth of 0 °C isotherm on 28 December 2019 at the foot of the left slope is about 2 m, which is basically consistent with the monitoring data in Figure 7. As shown in Figure 8b,d, with the continuous decrease of external temperature, the temperature of the left slope dropped to −15 °C in February 2020, and the freezing front continued to penetrate to the interior of the subgrade, so the depth of the 0 °C isotherm increased. Note that the depth of the 0 °C isotherm of the left shady slope was deeper than that of the right sunny slope, showing obvious sunny–shady slopes effect.

It was shown in Figure 8c that the simulated results matched well with the actual monitoring value of the underground temperature at the middle line. Both the simulated results and actual monitoring value show that the temperature near the subgrade surface is greatly affected by the external environment. However, the underground temperature is relatively stable when exceeding a certain depth.

As shown in Figure 8e, due to the continuous freezing time, the freezing front continued to penetrate the deep area, as the 0 °C isotherm depth reached about 2.7 m below the surface on 1 April 2020. At this time, the external temperature rose somewhat and the surface temperature of the subgrade was about −5.5 °C. As shown in Figure 8f, by 12 June 2020, the air temperature had risen to a positive temperature, and the internal temperature of the subgrade was lower than that of the outside, so heat was absorbed from outside to the inside subgrade, which caused the shallow ice layer to melt.

It can be seen from Figure 8c,d,g,h that the monitoring value fluctuates around the simulation value in a certain deviation. In the real world, the physical properties of soil are discreteness, heterogeneous, and spatial variability, which control the spatial variability of ground temperature distribution. As a result, the monitored values are not as smooth as the simulated values, and even show some unpredictable outlier points. However, the overall trend is consistent, indicating that it is a reasonable to apply the THM coupling model to highway subgrades in seasonally frozen regions.

#### 4.1.2. Spatial–Temporal Distribution of Moisture Field

The variation of moisture field of subgrade is also significantly affected by seasonal changes. The volumetric unfrozen water content changes greatly in the shallow range under the subgrade surface, which is manifested as a significant decrease in the negative temperature season and an increase in the positive temperature season.

**Figure 9:**
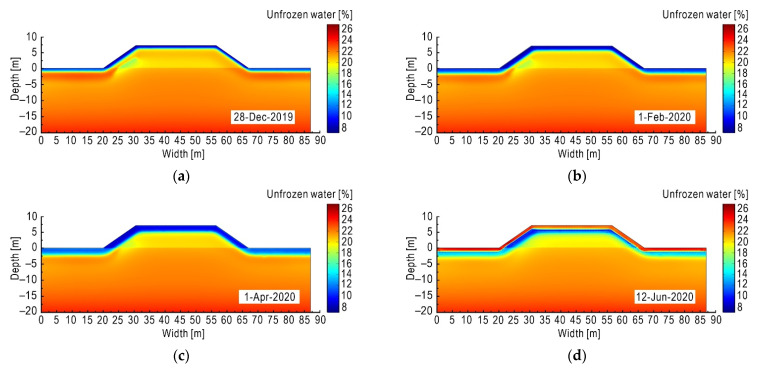
Subgrade moisture field at different times. (**a**) Volumetric unfrozen water content on 28 December 2019; (**b**) volumetric unfrozen water content on 1 February 2020; (**c**) volumetric unfrozen water content on 1 April 2020; (**d**) volumetric unfrozen water content on 12 June 2020.

As shown in Figure 9a–c, in December 2019, February 2020 and April 2020, the external temperature is negative. At these time points, most of the unfrozen water near the subgrade surface began to freeze, causing the volumetric unfrozen water content to drop sharply, which in turn caused an increase in suction. Driven by the suction, the unfrozen water from the nearby area migrates to the frozen area and then freezes. As temperatures are lower in the shady slope than in the sunny slope, more freezing occurs in the shady slope, resulting in more reduction in unfrozen water and more frost heave.

As shown in Figure 9d, in June 2020, as the external temperature gradually rises to a positive temperature, the melt starts from the subgrade surface and gradually extends to the interior of the subgrade. Following the ice melting process, the content of unfrozen water increases rapidly; however, at the same time, the ice inside the subgrade has not completely melted, and there still exists an area with low unfrozen water content inside the frozen area. Due to having experienced more freezing, the left slope (shady slope) contains more ice than the right slope (sunny slope). Again, due to the small amount of solar radiation received, the melting depth of the left slope is less than that of the right slope.

The difference between the sunny slope and the shady slope in the melting process and the asynchrony in time will aggravate thaw collapse. At the same time, the existence of freeze–thaw interface causes the stability of the subgrade to evolve in an unfavorable direction.

### 4.2. Analysis of Freeze–Thaw Damage of Subgrade

#### 4.2.1. Freeze–Thaw Damage of Original Subgrade (Without Anti-Frost Measures)

**Figure 10:**
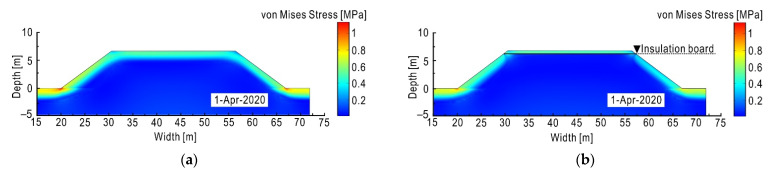

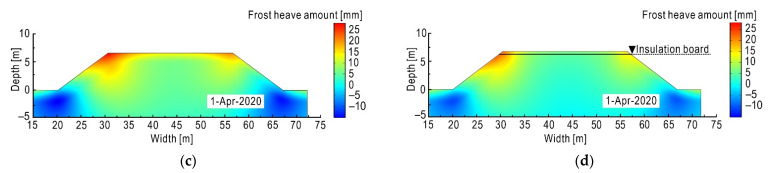
Frost heave characteristics of subgrade on 1 April 2020. (**a**) Von Mises stress distribution of original subgrade; (**b**) von Mises stress distribution of insulation board subgrade; (**c**) frost heave amount of original subgrade; (**d**) frost heave amount of insulation board subgrade.

Considering that as of April 2020, the subgrade has been frozen for enough time with a deep frost depth, so the time point of 1 April 2020 was selected for analysis. The physical fields of subgrade are shown in Figure 10a,c.

As shown in Figure 10a, the frost depth of subgrade is about 2.7 m from pavement, with the von Mises stress [58,69] mainly distributed in the shallow layer under the pavement. As the temperature drops, the shallow layer of the subgrade undergoes a freezing process, leading to frost heave, which causes an increase in frost heave stress. However, the soil in the deep layer of the subgrade is less frozen and that is why the stress keeps in a low level there. It should be noted that the frost heave stress is not directly displayed in Figure 10a, but the von Mises stress, which has a positive correlation with the former, alternatively illustrates the frost heave stress. This is mainly because the fact that the COMSOL does not directly provide the frost heave stress defined by Equation (5), while von Mises stress is accessible.

Although the frost heave occurs on both slopes and the pavement, it concentrates on the slope shoulder. Therefore, the deformation deduced by frost heave at the slope shoulder of the subgrade is significantly greater than that at the middle line of the subgrade. Due to the effect of sunny–shady slopes, the frost depth in the shady slope (left slope) is deeper. In winter, more water migrates there and then freezes, forming a thicker ice lens, which leads to a greater frost heave deformation at the left slope shoulder. When the temperature rises to positive, the phenomenon of frost nucleus becomes more significant, and then it does not drain well after thawing, which leads to an increase of soil moisture and a decrease of soil strength. Therefore, a large thaw settlement deformation occurs, and the left shaded slope suffers greater damage than the right sunny slope. The uneven deformation of subgrade due to the effect of sunny–shady slopes causes cracking damage to the pavement and affects the usability and safety of the highway.

**Figure 11:**
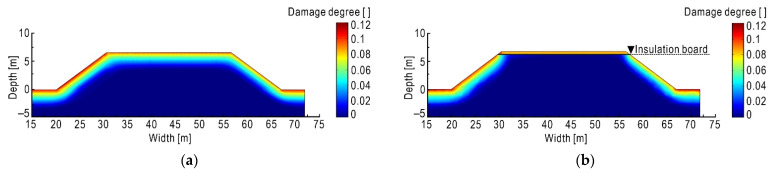
Contours of freeze–thaw damage degree of subgrade. (**a**) Original subgrade; (**b**) insulation board subgrade.

In terms of the quantitative characterization of subgrade damage, the new method in Section 2.3.2 is applied. Taking the period from 1 August 2019 to 1 August 2020 as the statistic window, the volumetric ice content in every 24 h of subgrade is derived and then its standard deviation is calculated, so as to characterize the subgrade damage caused by frost heave and thaw settlement in a full year. The results are shown in Figure 11a. The damage area caused by freeze–thaw cycles is mainly distributed near the surface of the subgrade. The closer to the surface of the subgrade, the larger the degree of damage because the surface of the subgrade is more susceptible to the environment temperature, which results in greater fluctuations of ice content within the full year.

#### 4.2.2. Freeze–Thaw Damage under Anti-Frost Measures

In the season of negative temperature, the subgrade will produce uneven frost heave, which threatens the safety and stability of pavement. The main ideas for preventing and mitigating frost damage are: (1) control the water content of subgrade at a low level, (2) reduce the influence of ambient temperature change, (3) reduce frost heave sensitivity of subgrade soil, and improve soil strength.

Measure 1 is to install an insulation board [70,71] with a thickness of 0.1 m at a depth of about 0.5 m below the pavement. The insulation board has a good thermal insulation performance, which can effectively block penetration of the freezing front so as to reduce the frost depth of the subgrade, thereby weakening the frost heave effect. In order to study the anti-frost heave performance of the insulation board, the frost heave amount of the subgrade installed insulation board was compared with that of original subgrade in the time point of April 2020. In addition, two extra working conditions are considered: (1) on February 2020 (the coldest month of the year), and (2) on the extreme cold weather (suppose −30 °C temperature lasts about 10 days), so as to compare the frost heave deformation and damage degree of the subgrade.

As shown in Figure 10b,d, the frost depth is significantly reduced compared to Figure 10a,c due to the thermal block effect of the insulation board. The distribution of ice in the subgrade is largely limited to the narrow area from the subgrade surface to the insulation board, thereby reducing the frost depth by about 2.2 m. Due to the reduction of frost depth, the melting depth of the subgrade will be correspondingly less than that of the original subgrade when the warm season comes, and the fluctuation of frost heave and thaw settlement of subgrade will be less, which is conducive to the stability of subgrade. Similarly, the von Mises stress distribution in Figure 10b is also hindered by the insulation board when comparing with Figure 10a, so that the stress distribution under the insulation board is maintained at a low level. As a result, the frost heave of the subgrade is significantly reduced by implementing measure 1.

Comparing Figure 11a,b, it can be seen that due to the thermal block effect of the insulation board, the freeze–thaw damage is greatly reduced and concentrated within a narrow area between the pavement surface and the insulation board. This phenomenon also indicates that if non-frost-susceptible soil (e.g., gravel, lime soil) is replaced between the insulation board and the road surface, it will further improve antifreeze effect.

**Figure 12:**
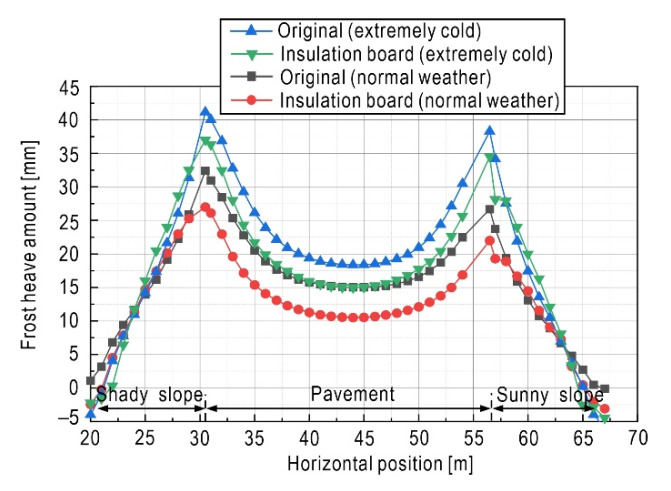
Frost heave amount of subgrade surface on 22 February 2020.

**Figure 13:**
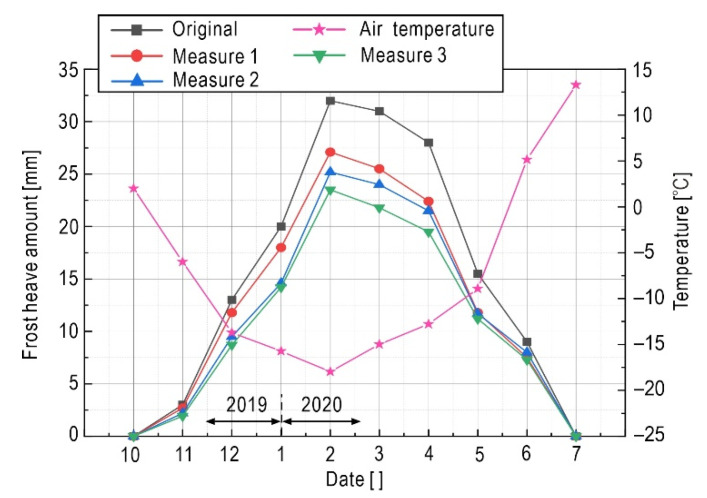
Frost heave amount time-history of left shoulder of subgrade under different anti-frost measures.

Taking time point of 22 February 2020 as an example, Figure 12 shows the distribution of frost heave amount of the surface. Results from two measures (original subgrade versus subgrade installed on insulation board) and two working conditions (normal weather versus and extremely cold weather) were illustrated. The frost heave amount on the surface of subgrade varies with the horizontal position, and presents a concave shape distribution. However, the distribution is not bilaterally symmetrical due to the effect of sunny–shady slopes, and the shoulder in the left shady slope produced a greater amount of frost heave than the right sunny slope did. Due to the existence of the insulation board, the frost heave amount of the pavement is obviously reduced, but because no insulation measures are set on both side slopes, the frost heave amount has little change compared with the original subgrade. Under the influence of extreme weather, the frost heave amount increases significantly. Both the shady slope and the sunny slope present a large deformation, which result in an extremely adverse impact on the stability of the subgrade. The insulation board reduces the impact of extreme weather to a certain extent, and the overall frost heave amount of the subgrade is reduced. Measure 2 is based on measure 1 by setting a cement stabilized sand structure layer [20] on the insulation board to enhance the anti-frost heave performance. Moreover, measure 3 supplements measure 2 by adding a waterproof layer of gravel [72] at the bottom of the subgrade (Measure 3). Under these three types of anti-frost heave measures, the frost heave displacement time-history on the left slope shoulder of the subgrade is shown in Figure 13, where the corresponding material parameters are listed in Table 8. The resulting peak value of frost heave displacement on the surface of the subgrade in the freezing period is shown in Table 9.

Regarding the time-history features shown in Figure 13, the frost heave of subgrade starts in October, and the maximum value appeared around February 2020. After June 2020, the amount of frost heave is not obvious. The overall process is increasing first and then decreasing within the statistic period. The maximum value of the left slope shoulder in the condition of the original subgrade is 31.5 mm, which is less than the allowable value specified in the Chinese design code [73] (allowable frost heave amount of the asphalt pavement highway subgrade is 40 mm). Meanwhile, it should be noted that this paper does not consider rainfall, water accumulated on the surface of the subgrade, and other conditions in the calculation. Therefore, the assumption of only focusing on the redistribution of the internal moisture of the subgrade without considering the external supplementary water will lead to an idealized result, and then underestimate the degree of damage to the subgrade.

Additionally, Figure 13 presents the anti-frost performance of different measures. In the condition of the insulation board (measure 1), the frost heave amount is weakened due to the heat block effect, and the maximum value of the left slope shoulder is reduced to 27.1 mm. In the condition of measure 2, the cement-stabilized sand structural layer has the characteristics of high structural strength, large stiffness and good water stability, which can improve the strength of the subgrade, so the corresponding maximum frost heave amount is reduced to 25 mm. In the condition of measure 3, because the subgrade with insulation board, cement stabilized sand structure layer and waterproof layer of gravel has the weakest frost heave effect, so the corresponding maximum frost heave amount decreases to about 23.5 mm. This shows that a mixed anti-freezing measure can effectively reduce frost heave, ensuring the stability of the highway in a seasonally frozen region. The resulting peak value of frost heave amount on the pavement in the full year (from 1 August 2019 to 1 August 2020) is shown in Table 9.

## 5. Conclusions

(1) Considering ice–water phase transition, convective heat transfer, and ice blocking effect, the THM coupling model of frozen soil was established based on the Harlan model, and then implemented by secondary development on COMSOL. The confirmative simulation for soil column freezing experiment verified the effectiveness of the THM coupling model.
(2) Based on the on-site monitoring data, the temperature field and moisture field of the seasonal frost subgrade of the Golmud-Nagqu section of National highway G109 are simulated. The results showed that both temperature and volumetric unfrozen water content had obvious seasonal variation characteristics, and there exist significant differences between the shady slope and the sunny slope. The overall trend of the simulated temperature values conforms well with the on-site monitoring data, so it is reasonable to apply the suggested THM coupling model to highway subgrade in seasonally frozen regions.
(3) Based on the THM coupling model, the frost heave effect of subgrade is simulated. It shows that the frost depth of subgrade in April is about 2.7 m from the surface. Due to the existence of sunny–shady slopes effect, both frost heave amount and frost depth are larger in the left shady slope shoulder than that of right sunny slope shoulder.
(4) The standard deviation of the volumetric ice content in the monitoring period is used to characterize the degree of freeze–thaw damage of the subgrade, which is simple and intuitive. The results show that the shallow layer of subgrade experienced more damage than the deep layer because the former is more easily affected by the external environment. The damage degree of the left shady slope is greater than that of the right sunny slope. By applying the insulation board, the damage area is greatly reduced and is concentrated between the subgrade surface and the insulation board.
(5) The frost heave characteristics of subgrade installed insulation board is compared with that of the original subgrade. The results in extreme cold weather show that the insulation board presents ideal performance in reducing the influence of ambient temperature, thereby reducing the frost heave of the subgrade, which is beneficial to the stability of the subgrade.
(6) In order to strengthen the anti-frost effect, two more protective measures can be added on the basis of the insulation board measures (i.e., adding a cement-stabilized sand structure layer and a waterproof layer of gravel). The results show that the frost heave of the subgrade will be effectively controlled under the combined measures. The maximum frost heave amount of pavement will be reduced from 31.5 mm to 23.5 mm (i.e., reduced by 25%), which significantly reduces the freeze–thaw damage of the highway, and is conducive to the safety and stability of the highway subgrade in the seasonally frozen region.

## Figures and Tables

**Table 1:** Literature comparison list.

Author	Comments	Subgrade Type
Liu et al. [14,15]	The enthalpy change method was used to deal with the phase change problem, while the change of water field was not considered.	Railway
Yuan et al. [16]	The thermal state of special roadbed was analyzed, while the change of water field was not analyzed.	Railway
Tai et al. [17]	Anti-frost heaving simulation was carried out, while the sunny–shady slopes effect was not analyzed.	Railway
Zhang et al. [18]	The temperature boundary condition considering the sunny–shady slopes effect was established, while the deformation under special working conditions was not analyzed.	Railway
Zhang et al. [19]	The three-dimensional numerical model of frozen soil subgrade was established, while only the change of temperature field was analyzed.	Railway
Li et al. [20]	The thermal–hydraulic–mechanical coupling model of subgrade was established, without considering the sunny–shady slopes effect.	Highway
Wang et al. [21]	The boundary conditions of temperature field considering solar radiation and convective heat transfer were established. Only the sunny–shady slopes effect of thermal–mechanical field was analyzed.	Highway
Zhang et al. [22]	The thermal state of insulation subgrade was analyzed, but the subgrade deformation was not analyzed.	Highway
Zhang et al. [23]	The sunny–shady slopes effect of subgrade was analyzed, and the relationship between rational height of subgrade and groundwater level was discussed.	Highway
Liu et al. [24]	The influence of geosynthetic clay liner on the water-heat field of subgrade was analyzed, but the deformation field of subgrade was ignored.	Highway

**Table 2:** Initial temperature distribution of sample.

***h*** **(cm)**	0.00	1.52	3.04	4.56	6.08	7.60	9.12	10.64	12.16	13.68
***T*** **(** **°C** **)**	11.42	15.48	16.36	16.75	16.84	16.89	16.84	16.90	16.79	16.41

**Table 3:** Hydraulic parameters of model for soil column freezing experiment.

*a*	*b*	*m*	*l*	*θ_sa_*	*θ_re_*	*T_f_* (°C)	*k_s_* (m·s^−1^)	*ρ_d_* (kg·m^−3^)
2.6	0.56	0.5	0.5	0.42	0.02	−0.15	4 × 10^−^^7^	1500

**Table 4:** Thermal parameters of soil column freezing experiment.

*C_sf_*^1^ (J·kg^−1^∙K^−1^)	*C_su_*^2^ (J·kg^−1^∙K^−1^)	*λ_sf_* (W∙m^−1^∙K^−1^)	*λ_su_* (W∙m^−1^∙K^−1^)	*L* (J·kg^−1^)	*ρ_i_* (kg·m^−3^)	*ρ_w_* (kg·m^−3^)
1371.45	1638.77	1.63	1.28	334,560	917	1000

^1^ Subscript *f* denotes frozen soil, ^2^ subscript *u* denotes unfrozen soil.

**Table 5:** Parameters of temperature boundary.

Position	$T_{0}({}^{\circ}C)$	$A_{0}({}^{\circ}C)$	φ
Natural ground	0.72	16	π/2
Shady slope	−0.2	16.6	π/2
Sunny slope	3.4	16	π/2
Pavement	2.5	16	π/2

**Table 6:** Thermal parameters of soil layer.

**Soil Layer**	$\rho_{d}\;(\mathrm{kg}\cdot m^{-3})$	$C_{sf}\;(J\cdot {\mathrm{kg}}^{-1}\cdot K^{-1})$	$C_{su}\;(J\cdot {\mathrm{kg}}^{-1}\cdot K^{-1})$	$\lambda_{sf}\;(W\cdot m^{-1}\cdot K^{-1})$	$\lambda_{su}\;(W\cdot m^{-1}\cdot K^{-1})$	$T_{f}\;({}^{\circ}C)$
Subgrade soil	2060	710	790	2.53	1.86	−0.56
Foundation soil	1540	730	840	2.08	1.86	−0.56

**Table 7:** Hydraulic characteristic parameters of soil layer.

Soil Layer	*a*	*b*	*m*	*l*	*θ_sa_*	*θ_re_*	$k_{s}(m\cdot s^{-1})$
Subgrade soil	0.66	0.61	0.14	0.5	0.4	0.05	2×10^−^^4^
Foundation soil	1	0.47	0.26	0.5	0.41	0.06	6×10^−^^6^

**Table 8:** Different material parameters.

Materials	λ	C	$\rho_{d}\;(\mathrm{kg}\cdot m^{-3})$
Insulation board	0.03	1250	30
Cement stabilized sand	1.41	920	2233
Waterproof layer of gravel	0.396	1245	1490

**Table 9:** Peaks of frost heave amount on pavement during freezing period.

Measures	Subgrade Type	the Peak Value of Frost Heave Amount (mm)
No measure	Original subgrade	31.5
Measure 1	Insulation broad subgrade	27.1
Measure 2	Insulation broad + cement stabilized sand subgrade	25
Measure 3	Insulation broad + cement stabilized sand + waterproof layer of gravel subgrade	23.5

## Data Availability

Not applicable.

## Notations

C(θ)	Volume heat capacity of soil [$J/m^{3}$]
t	Time [s]
L	Latent heat of ice-water phase transition [J/kg]
ρ, $\rho_{d}$	Natural density and dry density of soil [$kg/m^{3}$]
$\theta_{i}$	Volumetric ice content [ ]
θ	Total volumetric water content [ ]
$k\left(\theta_{u}\right)$	Permeability coefficient of unsaturated soil [m/s]
$B_{i}\left(T\right)$	Solid–liquid ratio [ ]
$P_{frozen}$	Frost heave stress [MPa]
$E_{T}$	Young’s modulus of soil [MPa]
$\varepsilon_{vol}{}^{T}$	Thermal expansion volumetric strain [ ]
$\varepsilon_{th}$	Thermal expansion linear strain [ ]
$A_{1}$,$B_{1}$	Parameters related to the Young’s modulus of soil [ ]
$T_{ref}$	Reference temperature of thermal expansion [°C]
$\eta \left(\theta_{i}\right)$	Frost heave coefficient [ ]
$k_{s}$	Saturated permeability coefficient of soil [m/s]
S	Relative saturation of soil [ ]
I	Blocking effect of pore ice in frozen area [ ]
$\lambda_{i}$	Thermal conductivity of ice [$W\cdot m^{-1}\cdot K^{-1}$]
$C_{s}$	Soil specific heat [$J\cdot kg^{-1}\cdot K^{-1}$]
K	Inherent permeability of unsaturated soil [$m^{2}$]
$\eta_{w}$	The dynamic viscosity of liquid water [$N\cdot s/m^{2}$]
g	The acceleration of gravity [$m/s^{2}$]
T	Temperature [°C]
$\lambda \left(\theta \right)$	Thermal conductivity [$W\cdot m^{-1}\cdot K^{-1}$]
$\rho_{i}$, $\rho_{w}$	Represent density of ice and water, respectively [$kg/m^{3}$]
$C_{w}$	Specific heat of water [$J\cdot kg^{-1}\cdot K^{-1}$]
v	Liquid water flux [m/s]
$\theta_{u}$	Volumetric content of unfrozen water [ ]
$D\left(\theta_{u}\right)$	Water diffusion coefficient [$m^{2}/s$]
$T_{f}$	Subgrade freezing temperature [°C]
$K_{T}$	Bulk modulus [MPa]
$\mu_{T}$	Poisson’s ratio of soil [ ]
$\varepsilon_{x}{}^{T},\varepsilon_{y}{}^{T},\varepsilon_{z}{}^{T}$	Thermal expansion linear strain in three axes directions, respectively [ ]
α	Thermal expansion coefficient [ ]
$A_{2}$,$B_{2}$	Parameters related to the Poisson’s ratio of soil [ ]
$V_{frozen}$	Frost heave amount [mm]
$c\left(\theta_{u}\right)$	Specific water capacity [$m^{-1}$]
$\theta_{re}$	Residual volumetric water content [ ]
$\theta_{sa}$	Saturated volumetric water content
$C_{i}$	Specific heat of ice [$J\cdot kg^{-1}\cdot K^{-1}$]
$\lambda_{w}$	Thermal conductivity of water [$W\cdot m^{-1}\cdot K^{-1}$]
$\lambda_{s}$	Soil thermal conductivity [$W\cdot m^{-1}\cdot K^{-1}$]
$K_{r}$	The relative permeability of the liquid [ ]
$P_{w}$	The pore water pressure [Pa]
h	The pressure head [m]
